# Transcript Profile Analyses of Maize Silks Reveal Effective Activation of Genes Involved in Microtubule-Based Movement, Ubiquitin-Dependent Protein Degradation, and Transport in the Pollination Process

**DOI:** 10.1371/journal.pone.0053545

**Published:** 2013-01-03

**Authors:** Xiao Hui Xu, Fang Wang, Hao Chen, Wei Sun, Xian Sheng Zhang

**Affiliations:** State Key Laboratory of Crop Biology, Shandong Key Laboratory of Crop Biology, College of Life Sciences, Shandong Agricultural University, Tai’an, Shandong, China; Institute of Botany, Chinese Academy of Sciences, China

## Abstract

Pollination is the first crucial step of sexual reproduction in flowering plants, and it requires communication and coordination between the pollen and the stigma. Maize (*Zea mays*) is a model monocot with extraordinarily long silks, and a fully sequenced genome, but little is known about the mechanism of its pollen–stigma interactions. In this study, the dynamic gene expression of silks at four different stages before and after pollination was analyzed. The expression profiles of immature silks (IMS), mature silks (MS), and silks at 20 minutes and 3 hours after pollination (20MAP and 3HAP, respectively) were compared. In total, we identified 6,337 differentially expressed genes in silks (SDEG) at the four stages. Among them, the expression of 172 genes were induced upon pollination, most of which participated in RNA binding, processing and transcription, signal transduction, and lipid metabolism processes. Genes in the SDEG dataset could be divided into 12 time-course clusters according to their expression patterns. Gene Ontology (GO) enrichment analysis revealed that many genes involved in microtubule-based movement, ubiquitin-mediated protein degradation, and transport were predominantly expressed at specific stages, indicating that they might play important roles in the pollination process of maize. These results add to current knowledge about the pollination process of grasses and provide a foundation for future studies on key genes involved in the pollen–silk interaction in maize.

## Introduction

Compatible pollen–stigma interactions are crucial steps for the success of seed production in flowering plants. Most plants have evolved strict and complex recognition systems to accept suitable pollen grains and reject incompatible ones to increase the rate of successful reproduction. In general, the pollen–stigma interaction can be divided into five steps: pollen adhesion, hydration, germination, pollen tube penetration into the stigma, and pollen tube growth in the style [1]. In wet-stigma species, some of the processes appear to be passive and largely unregulated; for example, pollen adhesion and hydration [2]. In contrast, all five stages are under active regulation in dry-stigma species [1]. Over the past two decades, significant progress has been made in elucidating the molecular events of the pollen–stigma interaction in self-incompatible (SI) pollination systems; however, the detailed molecular mechanisms of the interaction between female and male reproductive tissues in compatible pollination are still unclear.

The stigma, which is located at the top of the pistil, plays a central role in pollination. In compatible pollination, pollen adhesion activates a chain of reactions in the stigma, resulting in water flow from the stigma into the pollen grains. Following hydration, pollen grains germinate and pollen tubes emerge to penetrate the stigmatic cell walls [3], [4]. Cell wall-modifying enzymes secreted from the stigmatic papilla, such as cutinases and esterases, break down the stigmatic cuticle, allowing pollen tube penetration [5], [6], [7]. Knocked-out or reduced expression of *Exo70A1*, whose protein localized to the plasma membrane of the stigmatic papillae, led to poor pollen hydration and tube penetration in both *Arabidopsis* and *Brassica* [8]. Once pollen tubes penetrate into the stigma, various signaling molecules and chemical attractants in the transmitting tract serve as key regulators for pollen tube growth and guidance [9], [10], [11], [12].

High-throughput tools, such as microarrays and RNA-Seq, have been used to analyze gene expression in the pollen and stigma, the partners that participate in pollination in different species [13]–[25]. Recently, a transcriptome analysis of pollen tubes growing through the stigma and style revealed a dramatic and distinct gene expression profile compared with those of mature pollen grains and pollen tubes grown *in vitro* [26], indicating that pistil factors have significant effects on pollen tube growth. Furthermore, comparison of gene expression profiles among unpollinated pistils and pistils after pollination at different time points identified 1,373 genes that were differentially expressed during pollen–pistil interactions in *Arabidopsis* [27]. These studies are good examples of the importance of transcriptome analyses for studying pollen–pistil interactions.

Maize, one of the most important cereal crops in the world, has been used as a model species to study pollen–stigma interactions [28]. Like typical grasses, maize silk is twin-branched and covered with villous structures known as silk hairs, the function of which is equivalent to the stigmatic papillae in the Cruciferae. Different from eudicot species, maize silk is extraordinarily long, and contains two transmitting tracts distributed in both sides [29], [30]. Microarray and RNA-Seq technologies have been used to carry out transcriptome analyses of maize silk in different maize inbred lines B73 and Zheng58 [25], [31], [32].

Two groups of genes expressed in maize silk are most likely to participate in the pollen–silk interaction. One group includes genes that are expressed specifically or preferentially in maize silk, the other group includes those that are differentially regulated before and after pollination. In our previous study, 1,427 maize silk-specific/preferential genes were identified in the inbred line Zheng58 [25]. Here, the transcript profiles of maize silks were analyzed at different developmental stages representing the most important events during pollination. In total, 6,337 differentially expressed genes were identified, including both well-known and candidate genes involved in pollination. This study provides a new insight into the complex regulation networks underlying the pollen–silk interaction in maize during pollination.

## Results and Discussion

### RNA-Seq Analysis of Silks at Four Developmental Stages

**Figure 1:**
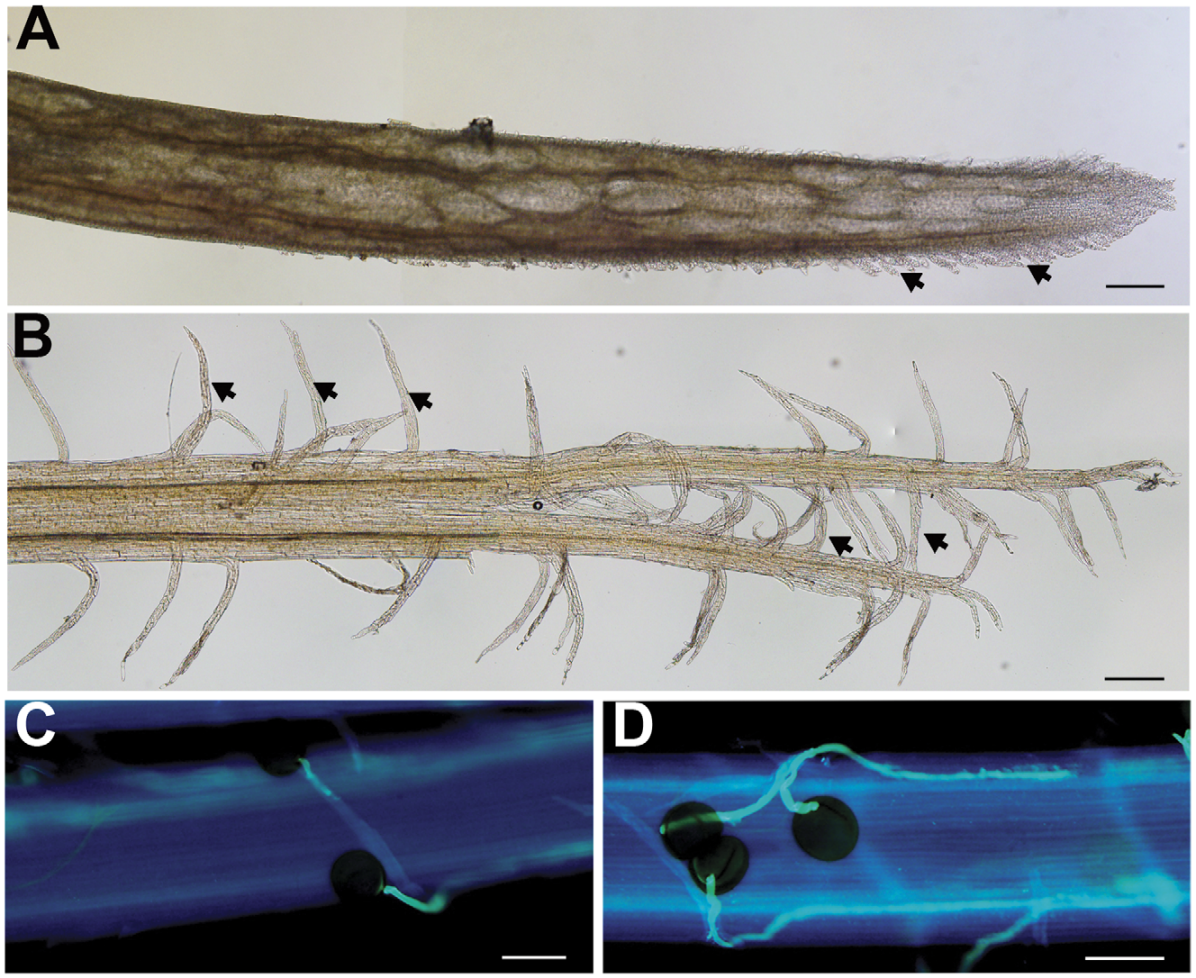
Maize silk tissues at four different developmental stages. (A) Immature silk. (B) Mature silk. (C) Silk at 20 min after pollination. (D) Silk at 3 h after pollination. Scale bars = 200 µm (A and B), 100 µm (C and D).

In maize, silk development can be divided into three stages according to pollen receptivity: immature partially receptive silks (total length 0–10 cm), mature fully receptive silks (total length 10–25 cm), and silks after pollination [33]. Fresh mature pollen grains adhered poorly to silks with total length less than 1 cm, and they could be washed away easily with water. These silks were defined as immature silks (IMS) (Figure 1A). In contrast, mature silks (MS) (Figure 1B) showed high affinity for mature pollens. Within the first 20 minutes after pollination (20MAP), most pollen grains hydrated and germinated on the silk hairs, and invaded the stigmatic tissues (Figure 1C). At 3 hours after pollination (3HAP), pollen tubes were growing inside the transmitting tracts (Figure 1D). The silk tissues at four stages represented three crucial steps of pollination: acquisition of pollen receptivity, pollen germination and penetration, and pollen tube growth and guidance. To identify genes involved in the pollination process, RNA-seq analyses were conducted for the four stages of maize silks. The isolated mRNAs from the four tissues were used to construct libraries, and were then sequenced by Illumina HiSeq™ 2000. After removing dirty raw reads, the number of filtered clean reads per library ranged from 6,145,170 to 7,374,812 (Table S1), a tag density sufficient for quantitative analysis of gene expression.

**Figure 2:**
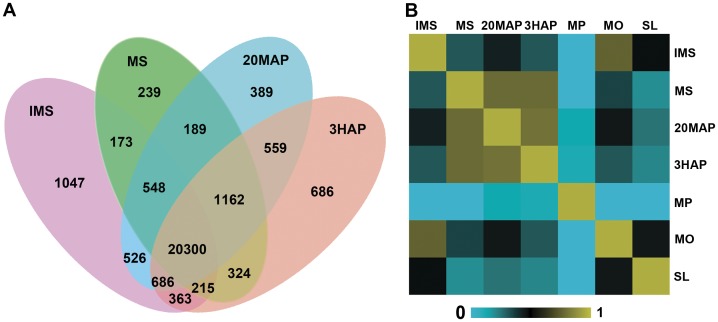
Gene expression of maize silks at four stages and correlation matrices of their RNA-seq libraries. (A) Distribution of genes expressed in four studied maize silk tissues. (B) Spearman correlation coefficient analysis of their RNA-seq libraries.

To identify genes corresponding to the reads in each library, the filtered clean reads were mapped to version 2 of the maize B73 reference genome (AGPv2, http://www.maizesequence.org) using Short Oligo-nucleotide Alignment Program 2 (SOAP2) aligner [34]. To ensure that the libraries were meaningful, reads that appeared only once were eliminated from further statistical analyses. The analysis was extended to study global patterns of gene expression during the four developmental stages of silks to find common and different characteristics. Both unique and overlapping genes expressed in the four samples were detected. In total, 23,858 (IMS), 23,150 (MS), 24,359 (20MAP), and 24,295 (3HAP) genes were detected (Figure 2A and Table S2). A total of 27,406 genes (69.1% of the filtered gene set of maize inbred line B73) were expressed during the whole process, of which 20,300 genes (74.1% of all the expressed genes in the four samples) were expressed at all four stages. Most of the genes, for example, 22,811 genes (95.6% of genes expressed in IMS) expressed in IMS were also expressed at one or more of the later stages, lending further support to each individual observation (Figure 2A).

The transcript profiles of mature pollen (MP), mature ovary (MO), and 6-day-old seedlings (SL) of the maize inbred line Zheng58 were sequenced by Xu *et al*. [25]. To establish the relationship between our experimental samples and those analyzed by Xu *et al*. [25], a Pearson correlation coefficient (PCC) analysis was performed on the sequencing libraries of the seven samples. As shown in Figure 2B, gene expression profiles in MS, 20MAP, and 3HAP showed high similarities, but they differed from that in IMS, which was consistent with their different responses when pollen grains landed on the silks. Surprisingly, the gene expression profile of IMS was similar to that of the mature ovary. This may be because these two tissues shared the same origin, or they are located adjacent to each other.

### Changes in Gene Expression Profiles among the Different Developmental Stages

To identify differentially expressed genes during pollination, the significance of digital gene expression analysis was carried out [35]. Three sets of data were compared: MS vs. IMS, 20MAP vs. MS, and 3HAP vs. 20MAP. Meanwhile, although as many pollen grains as possible were removed from the silk, a small quantity of pollen grains possibly remained in 20 MAP and 3 HAP silks because of pollen tube growth. For that reason, the MP transcript profile was used as a control. As pollen transcripts were highly diluted in 20MAP and 3HAP silks, they would have only minor effects on the transcription profiles of the two samples.

Using thresholds of fold change ≥2 and false discovery rate (FDR) <1E−10, 6,337 differentially expressed genes derived from at least one of the three comparisons were selected (Table S3). Among them, 291 genes were expressed in IMS but not in MS, suggesting that most of them might be involved in silk growth and development (Table S4A). There were 317 genes that were absent in IMS, but expressed in MS. These genes may be involved in stigma final differentiation and maturation to be ready for pollination (Table S4B). In addition, there were 172 genes that only expressed in 20MAP and/or 3HAP silks, but not in IMS, MS, and MP (Table S4C), indicating these gene expression was up-regulated during pollination. We defined the 172 genes as genes which were induced by pollination.

**Figure 3:**
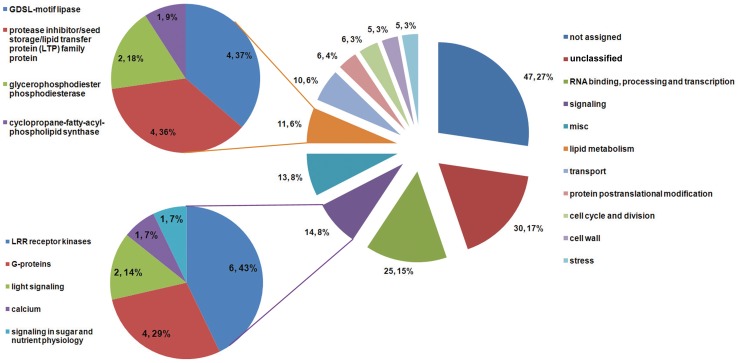
Classification of genes which were induced by pollination. 172 genes which were induced by pollination were classified into 11 categories by MapMan. Details of genes involved in signaling and lipid metabolism are shown on left.

To understand the functions of the genes which were induced by pollination, the genes were classified into 11 functional categories according to MapMan annotation [36]. As shown in Figure 3, the largest category consisted of genes without annotation (27%), while most of the well-annotated genes were involved in RNA binding, processing and transcription (15%), signaling (8%), and lipid metabolism (6%).

Among the 25 genes involved in RNA binding, processing and transcription, 15 genes encode transcription factors, and they distributed in various subfamilies, such as MYB, AP2/EREBP, C2H2, B3, C2C2-GATA, etc. (Table S4C). Many members of these subfamilies have been identified to play important roles in reproductive processes [37], [38], [39], [40]. For example, MYB transcription factors were found to regulate female reproductive organ development and pollen tube guidance [39], [40]. Considering that the expression of transcription factor genes is regulated by pollination, we suggest that these genes function in the process of pollen-silk interactions in maize.

Leucine-rich repeat receptor-like protein kinases (LRR-RLKs), the largest subfamily of plant RLKs, are involved in cell-to-cell signaling interactions [41], [42]. Pollination-induced genes encoding LRR-RLKs constitute the largest proportion in the signaling category (Figure 3). Previously, two LRR-RLKs (LePRK1 and LePRK2) localized to growing pollen tubes were identified as activated signal transducers in tomato [43], [44]. These kinases could interact with different extracellular ligands at different stages of the pollen–pistil interaction to perceive and transduce extracellular cues. For example, the extracellular domain of LePRK2 interacted with the pollen-specific protein LAT52 *in vitro* [45]. Considering the essential roles of LAT52 in pollen hydration, germination, and pollen tube growth [46], [47], this finding suggested that the formation of the LePRK2–LAT52 complex may be required to regulate pollen development on the stigma [45]. In addition, another secreted cysteine-rich protein, LeSTG1, which specifically accumulated in the stigma and style, not only could interact with the extracellular domain of LePRK2, but could also bind to the extracellular domain of LePRK1 [48]. The appearance of LeSTG1 could displace LAT52 to interact with LePRK2. A previous study also showed that exogenous LeSTG1 significantly promoted pollen tube growth. The concomitant formation of the LePRK1–LeSTIG1 and LePRK2–LeSTG1 complexes may be a checkpoint for pollen tube growth during the pollen–stigma interaction [49]. In the present study, genes involved in signaling accounted for 8% of all genes whose expression was induced by pollination. There were six genes encoding LRR-RLKs, and of them, one gene (*GRMZM2G465771*) encoding the protein showed moderate similarity (about 41%) to both LePRK1 and LePRK2. Interestingly, two genes in the differentially expressed gene set (*GRMZM2G317406* and *GRMZM2G165506*) were homologous to *LeLAT52* (Table 1), suggesting that interactions between LRR-RLKs and LAT52 might play important roles during pollination in maize.

**Table 1:** Genes show high similarity to well-known pollen–pistil interaction-related genes in the SDEG dataset.

Gene ID (maize)	Homologous gene	Description	Biological function	Reference
GRMZM2G377615	AT1G79860	Nucleotide exchange factor 12 (ROPGEF12) [*Arabidopsis thaliana*]	Pollen tube growth	[80]
GRMZM5G817886	AT3G22200	Pollen-pistil incompatibility 2 (POP2) [*A. thaliana*]	Pollen tube growth and guidance	[11]
GRMZM2G093900				
GRMZM2G004012	AT2G02850	Plantacyanin [*A. thaliana*]	Pollen tube penetration and growth	[10]
GRMZM2G445602	AT2G26250	Fiddlehead (FDH) [*A. thaliana*]	Pollen hydration and germination	[81]
GRMZM2G131026	AT3G04080	Apyrase 1 (AtAPY1) [*A. thaliana*]	Pollen germination	[82]
GRMZM2G178958	AT2G24200	Leucyl aminopeptidase 1 (LAP1) [*A. thaliana*]	Pollen adhesion	[83]
GRMZM2G075255	AT1G02205	CER1 [*A. thaliana*]	Pollen hydration	[84]
GRMZM2G099097				
GRMZM2G083526	AT5G57800	CER3 [*A. thaliana*]	Pollen hydration	[84]
GRMZM2G114642				
GRMZM2G029912				
GRMZM2G409312	AT1G68530	CER6 [*A. thaliana*]	Pollen hydration	[84]
GRMZM2G168304				
GRMZM2G062718				
GRMZM2G164974				
GRMZM2G070305	AT5G08470	Peroxisome 1 (Pex1) [*A. thaliana*]	Pollen tube growth	[85]
GRMZM2G381395	AT1G68990	Male gametophyte defective 3 (MGP3)[*A. thaliana*]	Pollen tube growth, femalegametogenesis and embryogenesis	[86]
GRMZM2G461279	AT3G59530	Leucyl aminopeptidase 3 (LAP3) [*A. thaliana*]	Pollen adhesion	[87]
GRMZM2G116010	AT3G56960	Phosphatidyl inositol monophosphate 5 kinase 4 (PIP5K4) [*A. thaliana*]	Pollen tube growth	[88]
GRMZM2G107839	AEV12221.1	Stigma/stylar cysteine-rich adhesion (SCA)[*Lilium longiflorum*]	Pollen tube growth and guidance	[52], [89]
GRMZM2G101958	AFD32273.1	Stigma/stylar cysteine-rich adhesion (SCA)[*Lilium* hybrid cultivar]	Pollen tube growth and guidance	[52], [89]
GRMZM2G010868	AFD32276.1	Stigma/stylar cysteine-rich adhesion (SCA)[*L.* hybrid cultivar]	Pollen tube growth and guidance	[52], [89]
GRMZM2G126397	AEV23221.1	Stigma/stylar cysteine-rich adhesion (SCA)[*L. longiflorum*]	Pollen tube growth and guidance	[52], [89]
GRMZM2G004012	AAR84219.1	Chemocyanin [*L. longiflorum*]	Pollen tube guidance	[90]
GRMZM2G023847				
GRMZM2G160370	AAB97738.1	Arm repeat containing protein [*Brassica napus*]	Self-incompatibility	[58], [60], [91]
GRMZM2G027375	ABY58019.1	Arm repeat containing protein 1 (ARC1)[*Brassica oleracea var. acephala*]	Self-incompatibility	[58], [60], [91]
GRMZM2G019777				
GRMZM2G125034				
GRMZM2G471733	AAB97738.1	Arm repeat containing protein [*B. napus*]	Self-incompatibility	[58], [60], [91]
GRMZM2G351387				
GRMZM2G452016				
GRMZM2G317406	CAA33854.1	Late anther tomato 52 (LeLAT52) [*Solanum lycopersicum*]	Pollen hydration, germination and growth	[47]
GRMZM2G165506				

Genes encoding GDSL-motif lipases and lipid transfer proteins were overrepresented in the category of lipid metabolism (Figure 3). Recent studies showed that these two subfamilies of genes related to lipid metabolism participated in regulating pollen–pistil interactions. The extracellular lipase EXL4, which is similar to GDSL-motif lipases, is required for efficient pollen hydration in *Arabidopsis* [50]. In lily, a wet-stigma species, the SCA lipid-transfer protein is abundant in the stigma and the transmitting tract, and is responsible for pollen tube growth and guidance [51], [52], [53].

### Bioinformatic Analyses of Genes Differentially Expressed During Pollination

**Figure 4:**
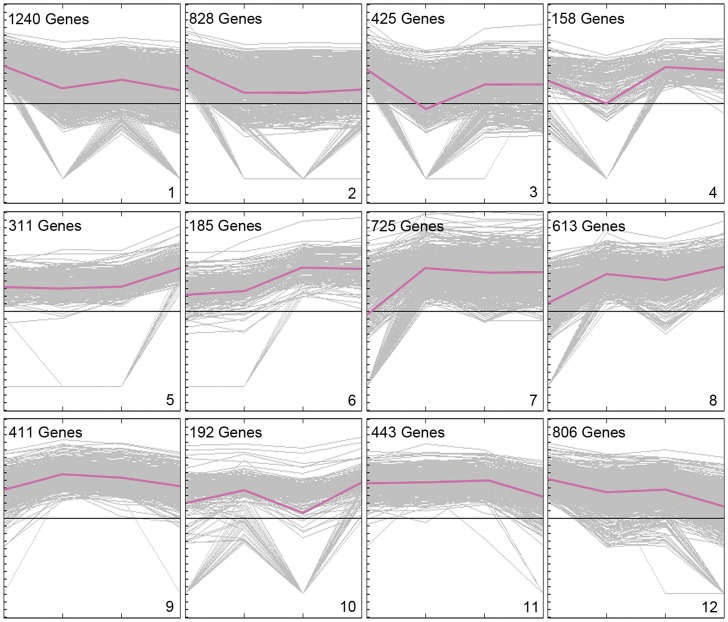
Clustering analysis of the differentially expressed genes in maize silk before and after pollination. 6,337 differentially expressed genes were clustered into 12 clusters by MEV 4.0. Number of genes in each cluster is shown at the top of each cluster.

The 6,337 differentially expressed genes in silks (SDEG) were grouped into 12 distinct clusters (K1–K12) based on their expression patterns using the K-means clustering algorithm (Figure 4). The genes in each cluster were listed in Table S5. The time-course clustering revealed that significant transcriptional changes occurred during the processes. The genes in K2, K5, K7, and K11 were differentially regulated at only one time point. Meanwhile, genes in the other clusters were differentially regulated at two or more time points (Figure 4). For example, genes in K3 (425 genes) showed a biphasic regulation–transcripts were down-regulated in MS but up-regulated at 20MAP. The expression levels of K2 (828 genes) and K7 (725 genes) showed significant differences from IMS to MS, indicating that they might be involved in silk growth and development. The 311 genes in K5 and 443 genes in K11 were specifically up-regulated or down-regulated from 20MAP to 3HAP, implying that these genes are involved in many developmental events occurred in silk, such as the programmed cell death of silk, and pollen tube growth following pollination. The transcript abundance of genes in K1 and K10 varied over the course of silk development and pollination.

**Figure 5:**
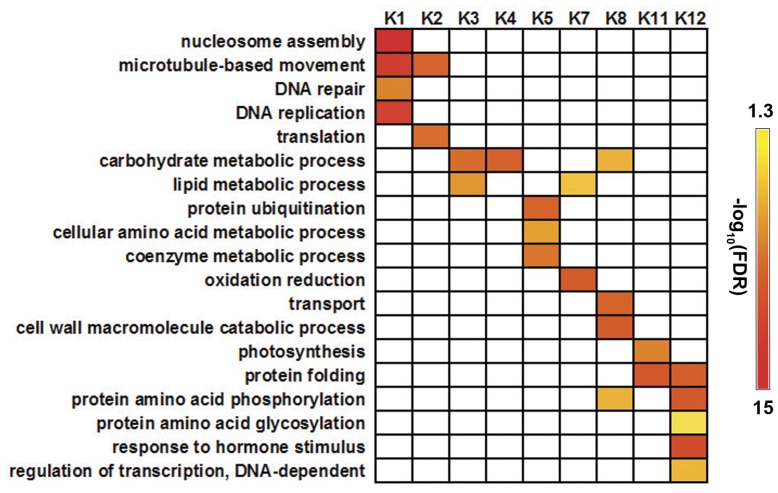
GO analysis of differentially expressed genes in each cluster. Using FDR<0.05 as the criterion, overrepresented GO terms (biological process) in each cluster were selected using the agriGO analysis tool. K1–K12 represents clusters 1 to 12, respectively.

GO analysis [54] was conducted on each cluster to distinguish differences among the 12 clusters. Because there were many novel genes classed into K6, K9, and K10, enriched GO terms could not be obtained for these three clusters. As shown in Figure 5, genes in other clusters showed distinct enrichment of GO terms, revealing that genes with different expression patterns might play different roles in the pollination process.

### Genes Involved in Microtubule-based Movement Might Regulate Silk Maturation and Pollen–silk Interactions

Microtubules (MT), the main dynamic structural components of the cytoskeleton, function in various aspects of cell physiology, such as development and maintenance of cell shape, cell motion and division, cell signaling, and intracellular transport [55]. A recent study showed that SI pollination led to moderate changes in MT organization, while compatible pollination resulted in a more severe localized depolymerization of MTs in *Brassica*. Further pharmacological, cell-biological, and genetic analyses showed that MT depolymerization in the stigmatic papilla is required for acceptance of compatible pollen [56].

In SDEG dataset, genes involved in MT-based movement were greatly enriched in K1 and K2, especially in K1 (Figure 4). There were 56 genes related to cytoskeleton in K1, 22 of which were involved in MT-based movement (Table S5). Their transcripts were down-regulated from IMS to MS, and up-regulated after pollination (Figure 4). The special expression patterns of MT-related genes indicated that MT-based movement is important in the process of silk maturation and pollination.

### Genes Involved in Ubiquitin-mediated Protein Degradation Likely Regulate Pollen Tube Growth and Development

Genes in K5 did not appear to show any changes in expression patterns in the first two processes, but their transcripts were predominantly up-regulated at 3HAP (Figure 4), suggesting that they may perform specific functions in regulating pollen-silk interactions. GO analysis revealed that genes with roles in protein ubiquitination, cellular amino acid metabolic process, and coenzyme metabolic process were well represented in this cluster (Figure 5). Among them, protein ubiquitination was the most significantly overrepresented GO term. MapMan analysis showed that all of the 13 genes associated with protein ubiquitination in K5 encoded Ring-type E3 ubiquitin ligases (Table S5), which are crucial for recognition of specific target proteins in ubiquitin-proteasome system (UPS).

Recent studies indicated that UPS plays important roles in SI. In sporophytic SI, Arm-Repeat Containing-1 (ARC1) with U-box-dependent E3 ubiquitin ligase activity is required for the stigma to reject self pollens. During this process, the landing of pollen carrying a cognate pollen-specific *S* locus protein 11 (SP11) on the stigma causes the phosphorylation and recruitment of ARC1. Then, numerous targets located in the stigma can be ubiquitinated and degraded by the 26S proteasome to prevent pollen germination [57], [58]. The substrates targeted by ARC1 are most likely compatibility factors. One of its downstream targets, Exo70A1, a putative subunit of the exocyst complex, was found to be an essential component for pollen hydration, germination, and pollen tube penetration in both compatible and SI pollinations via regulating or targeting vesicle trafficking to the plasma membrane [8]. Those findings suggested that ubiquitination by ARC1 affects regulation of the trafficking of vesicles containing growth-promoting materials. Thus, once SI pollination occurs, the trafficking will be blocked, and self pollen will be rejected [58], [59], [60], [61]. In gametophytic SI, the pollen *S* determinant F-box protein SFB (*S*-haplotype-specific F-box protein) or SLF (*S* locus F-box protein) is a subunit of the E3 ubiqutitin ligase SCF complex. In compatible interactions, cytotoxic pistil *S* determinant *S*-RNases can be recognized and ubiquitinated by a SCF^SFB/SLF^ complex, and then degraded via the 26S proteosome, allowing non-self pollen tube growth. In incompatible interactions, S-RNases can not be recognized, leading to inhibition of pollen tube growth [62], [63], [64]. Ubiquitin-mediated protein degradation also plays a role in animal fertilization and gametogenesis [65]. For example, there is an ubiquitin-dependent, sperm quality control mechanism in the mammalian epididymis. The surfaces of defective sperms bind ubiquitin and are then secreted into the epididymal epithelium. Then, defective sperms are phagocytosed by the epididymal epithelial cells [66]. It is likely that proteins labelled by ubiquitins are digested into small peptides that can be reused as new sources of nutrition for growing pollen tubes.

In our previous analysis, 61 genes encoding all of the components of the UPS system, including E1, E2, E3, and the 26S proteasome, were well represented in the MS-specific/preferential dataset [25]. In this study, 200 genes encoding UPS components were identified in the SDEG dataset (Table S5). Although their distributions were similar in both the MS-specific/preferential dataset and the SDEG dataset, the overrepresented Ring-type E3 ubiquitin ligase related genes (90 genes) and F-box related genes (39 genes) were much more abundant in the SDEG dataset than in the MS-specific/preferential dataset. Furthermore, genes related to ubiquitin-mediated protein degradation were significantly enriched in cluster K5 (Figure 5), and their expression levels were significantly up-regulated only from 20MAP to 3HAP (Figure 4). There was strong enrichment of UPS-related genes both in the MS-specific/preferential dataset and the differentially expressed gene dataset of pollination in maize silks. These findings suggested that UPS proteins, especially E3 ubiquitin ligases, are likely involved in the pollen–stigma interaction in self-compatible plants such as maize.

### Genes Involved in Transport Might be Required to Supply Abundant Nutrition to Guarantee Rapid Pollen Tube Polar Growth

**Figure 6:**
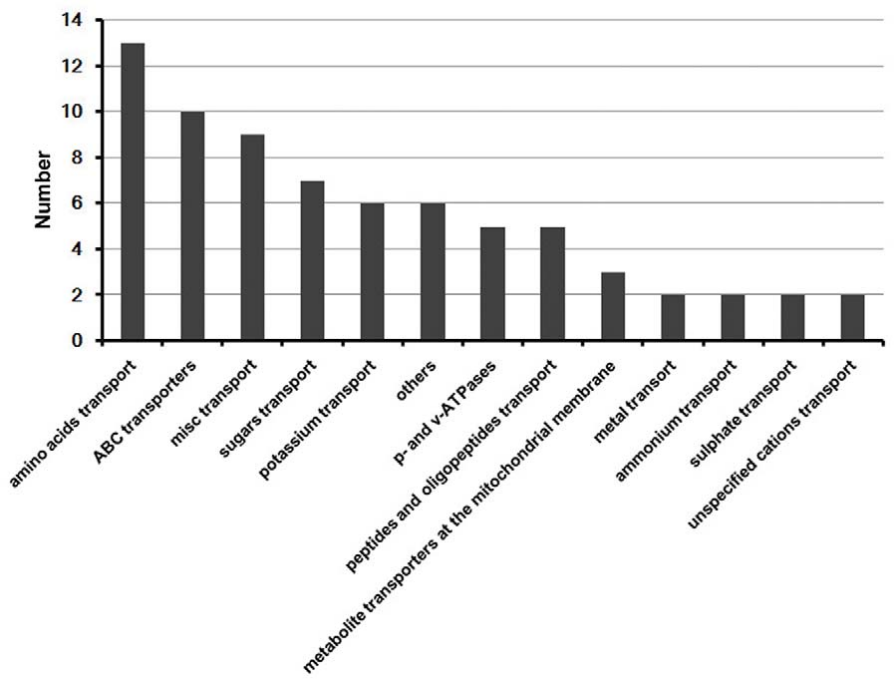
Genes involved in transport in cluster 8.

The expressions of genes in K8 (613 genes) were significantly up-regulated from IMS to MS, slightly down-regulated from MS to 20MAP, and then increased from 20MAP to 3HAP. This expression pattern suggested that they were consistently required for the silk to acquire its ability to accept pollen grains and support pollen tube growth and guidance *in vivo* (Figure 4). GO analysis showed that genes involved in transport, cell wall macromolecule catabolic process, carbohydrate metabolic process, and protein amino acid phosphorylation were strongly enriched in this cluster (Figure 5). Transport-related genes, in particular, genes encoding amino acid-, peptide-, and oligopeptide-transporters were overrepresented in the MS-specific/preferential dataset [25]. To find the overlaps between the two studies, MapMan was used to identify the genes involved in transport in K8. We found that gene terms related to amino acid-transporters and ABC transporters were overrepresented, while genes encoding peptide- and oligopeptide-transporters were not enriched (Figure 6 and Table S5).

Amino acid transporters direct the flow of amino acids, which are required for synthesis of new proteins to guarantee pollen tube elongation in the style [9], [67], [68], [69]. In *Arabidopsis*, one amino acid transporter gene, *Lysine/Histidine transporter 5* (*LHT5*), was not only expressed in germinating pollen during growth in the pistil, but also in the transmitting tract, implying that pollen tube growth in pistil requires active amino acid uptake [67]. Another amino acid transport-related gene, *LHT6,* was also expressed in the transmitting tissue, possibly serving the same function as *LHT5*. Recent study showed that D-serine formed in the pistil could modulate the activities of glutamate receptor-like proteins (GLRs), which regulate the cytosolic [Ca^2+^]_cyt_ in the pollen tube [70]. Because of the highly expression of amino acid transporter genes in the transmitting tract, these transporters might be responsible for amino acid uptake from the silk to the pollen tubes to support tube growth.

The second largest subfamily of transport-related genes in K8 was the ABC transporter subfamily (Figure 6 and Table S5). Members of this subfamily can transport a variety of biological molecules, such as lipids, sugars, polysaccharides, steroids, metal ions, inorganic acids, and glutathione conjugates across both extra- and intracellular membranes [71], [72]. To date, none of the mutants with defects in the synthesis of these proteins have been found to participate in the pollen–pistil interaction. However, spatial expression pattern analysis in tobacco identified a gene encoding a WBC subfamily ABC transporter, *NtWBC1*, which was preferentially expressed in the stigmatic secretory zone [73]. The stigmatic secretory zone is the first female tissue interacting with pollen grains and the one through which pollen tubes grow in the pistil. Therefore, this gene might play an important role in the pollen–pistil interaction in tobacco. Most of the ten genes encoding ABC transporters in K8 showed similar expression patterns to that of *NtWBC1*: six were expressed at high levels in MS but not in MP, three were strongly expressed in MS and weakly expressed in MP, and one was preferentially expressed in MP (Table S3).

Because maize silk is much longer than the stigmas of other eudicot and monocot plants, it requires more nutrition and guidance cues transported from the silk to maintain the rapid polar growth of pollen tubes [74]. Consistent with the results of the MS-specific/preferential dataset published recently [25], transport-related genes were overrepresented in SDEG dataset. Different from our previous findings, genes related to peptide- and oligopeptide-transporters were not enriched in K8, but genes encoding ABC transporters were overrepresented (Figure 6 and Table S5). This may be due to the more diverse substrates transferred by ABC transporters. Some of these substrates, such as lipids and metal ions, are important factors for regulating pollen–stigma interactions [75], [76], [77]. Besides, other transport-related genes, such as those encoding sugar transporters, and potassium channels were also well represented (Figure 6). Considering the wide range of substrates transported by these transporters and the morphological characteristics of maize silk, these findings suggest that genes related to transport, especially amino acid transporters and ABC transporters, function to supply sufficient materials from maize silk to the growing pollen tubes.

### Validation of RNA-Seq Results

Two methods were used to confirm the accuracy of the RNA-Seq data. First, the differentially expressed genes identified in previous studies (both transcriptome studies and those on expressions of single genes) and in the present study were compared to identify overlapping genes that participate in the pollen–silk interaction. Second, quantitative reverse-transcription PCR (RT-qPCR) analysis of the differentially expressed genes was conducted to confirm their expression patterns in the four samples used in this study.

In *Arabidopsis*, 1,373 differentially regulated genes that may be involved in the pollen–pistil interaction have been identified by microarray analysis [27]. By comparing the 6,337 genes in maize with the 1,373 genes in *Arabidopsis*, we found that a large number of genes differentially expressed in maize have no orthologs in *Arabidopsis*, suggesting that the mechanisms in pollen-stigma interactions exhibits some differences between eudicots and monocots. Meanwhile, we found that 397 genes in the SDEG dataset hit 276 differentially expressed genes in *Arabidopsis* (Table S6). The conserved differentially expressed genes, which may have similar functions during pollination in maize and *Arabidopsis*, could be used to verify the RNA-Seq results. GO analysis of these conserved genes revealed that genes involved in DNA replication, lipid metabolism, and amino acid metabolism were overrepresented, implying that they are conserved in the reproductive processes of both maize and *Arabidopsis* (Table S7). Sequence comparison also revealed that a number of the differentially expressed genes in our dataset showed high similarities to some well-known genes related to the pollen–pistil interaction identified in both dry- and wet-stigma species (Table 1). These genes and their homologs probably function in similar ways in the pollen–pistil interaction.

**Figure 7:**
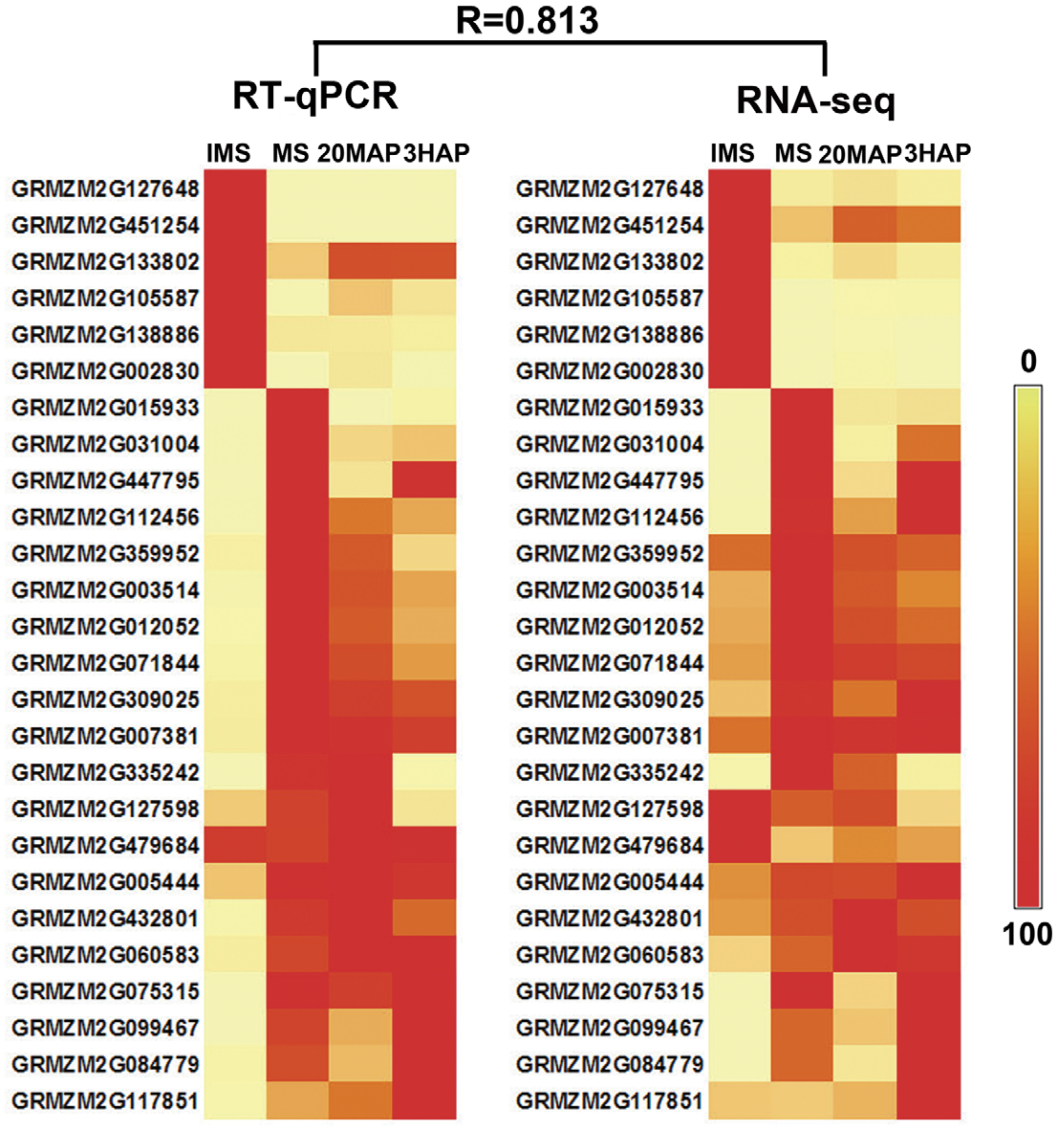
Validation of RNA-seq results by RT-qPCR. Expression levels of 26 randomly selected genes in the four samples used in this study were detected by RT-qPCR. Heat map (left) was constructed using mean values of expression levels derived from three biological replicates. Normalized RPKM values of RNA-seq results are shown (centre). For each gene, the maximum expression in a certain sample was set to 100, and relative expression levels in the other samples were calculated according to this maximum level. Relative expression is shown by colour scales as indicated on right. R represents the correlation coefficient value between the two platforms.

The expressions of 26 randomly selected genes were analyzed by RT-qPCR to validate the expression profiles obtained by RNA-Seq (Table S8). The expression patterns obtained by RT-qPCR were strongly correlated with the RNA-seq results (R = 0.813), supporting the reliability of the RNA-seq data (Figure 7).

Overall, these RNA-Seq results reflected dynamic gene regulation during silk development and the pollen–silk interaction, and provided a reliable dataset to identify candidate genes involved in pollination.

## Materials and Methods

### Plant Materials and Growth Conditions

The maize inbred line Zheng58 was grown in the field of Shandong Agricultural University Experimental Station, Tai’an, China. Ears in which the longest silk was less than 1 cm were regarded as immature. Silks in the centre of the ear were collected as IMS. Both tassels and ears were bagged before pollen shedding to avoid injury and cross-pollination. MS were collected as described by Xu *et al*. [25]. To collect pollinated silks, each cob with mature silks was evenly pollinated with approximately 0.3 g fresh mature pollen grains. After shaking to remove pollen grains that were not firmly adhered to MS, the upper parts of silks (approx. 4 cm) were collected at 20 minutes after pollination and 3 hours after pollination. To reduce biological variation, each experimental material was a mixture from at least three plants.

### RNA Extraction and Library Construction

Total RNA was extracted according to the modified CTAB protocol [78] and purified using an RNeasy MinElute Cleanup Kit (Qiagen, Valencia, CA, USA). The RNA concentration and purity was quantified by Nanodrop spectrophotometry (Nanodrop Technologies, Wilmington, DE, USA), and integrity was confirmed by an Agilent 2100 Bioanalyzer (Agilent Technologies, Palo Alto, CA, USA).

All four RNA-seq libraries were constructed from 5 to 8 µg total RNA. First, poly(A) containing RNA was isolated using oligo(dT) magnetic beads (Illumina, San Diego, CA, USA). Second, fragmentation buffer was used to break the mRNA into short fragments (approx. 200 bp). Third, the fragments were used as templates to synthesize first-strand cDNA using random hexamer-primers. Then, buffer, dNTP, RNase H, and DNA polymerase I were added to synthesize the second-strand cDNA. The double-stranded cDNA was purified using a QiaQuick PCR purification kit (Qiagen, Valencia, CA) and washed with EB buffer for end repair and single nucleotide adenine addition. Finally, the fragments were ligated to sequencing adaptors. Ligation products were size-selected by electrophoresis and the required fragments were enriched by PCR amplification. The quality and quantity of all libraries were assessed by Nanodrop ND-1000 spectroscopy (Thermo Scientific, Waltham, MA) and with an Agilent 2100 Bioanalyzer.

### Illumina Sequencing and Data Analysis

Libraries of each sample were sequenced using the Illumina HiSeq 2000 at Beijing Genomics Institute (BGI; Shenzhen, China). Single-end 49-bp reads were collected. Raw reads were derived from original image data, which were transformed into sequence data via base calling. The sequencing quality was analyzed by the Illumina Genome Analysis Pipeline version 1.6 software package. Dirty raw reads, including low quality reads, reads with more than 10% unknown bases, and reads with adaptors were removed. The remaining reads were defined as filtered clean reads. All sequence data has been submitted to ArrayExpress database under accession number E-MTAB-964.

Filtered clean reads were then aligned to the AGPv2 maize B73 reference genome through SOAP2 [34]. Mismatches of no more than two bases were allowed in the alignment. According to the results of the alignment, clean reads were subdivided into unique matched reads, multi-position matched reads, and unmapped reads. Only the unique matched reads were used to calculate the digital gene expression levels. The raw digital gene expression counts were normalized using the RPKM method [79]. If a gene had more than one transcript, the longest one was used to calculate its expression level and coverage.

To better understand the properties of the experimental samples, the sequencing data from MP, MO, and SL were extracted [25]. Then, a PCC analysis was conducted on all the seven samples used in both the two studies through the R package. To reduce sequencing errors, genes with fewer than two clean reads were omitted. The log_2_-transformed RPKM values of genes expressed in at least one of the seven samples were used for PCC analysis. The expression value of genes with log_2_-transformed RPKM values less than zero were set to zero. The heat map showing the correlation values of the seven tissues was drawn by Scalable Vector Graphics.

To identify differentially expressed genes in the whole process, the significance of digital gene expression analysis was tested [35]. Using fold change ≥2 and FDR ≤1E−10 as the criteria, the genes differentially expressed in at least one of the three comparisons (MS vs. IMS, 20MAP vs. MS, and 3HAP vs. 20MAP) were regarded as differentially expressed genes during pollination.

Gene annotations were derived from AGP v2 5b.60 (http://www.maizesequence.org/index.html). According to MapMan annotation, genes differentially expressed during the process were classified into various categories. GO analysis was performed using the Singular Enrichment Analysis tool (http://bioinfo.cau.edu.cn/agriGO/analysis.php). Clustering of the differentially expressed genes during pollination was performed using the K-Means/K-Medians Support module in MEV v4.8 (http://www.tm4.org/mev).

Two statistical analyses were conducted to find the overlaps between our SDEG dataset and the pollination-regulated genes identified by Boavida *et al*. [27] in *Arabidopsis*. First, the protein sequences of all the 6,337 differentially expressed genes were extracted and used as queries to blast against the TAIR 10 *Arabidopsis* protein database (http://www.arabidopsis.org/Blast/index.jsp). Using E-value ≤1e−10 as the cutoff, the best hit *Arabidopsis* gene was considered as the homolog of each maize gene. Second, the homologous genes with identical locus names to those of identified by Boavida *et al*. [27] were selected. Also, the protein sequences of well-known pollen–pistil interaction-related genes were extracted and then used to blast against the maize APGv2 5 b filtered gene set peptide database (http://www.maizesequence.org/blast) to find candidate functional genes in the maize pollen–pistil interaction.

### RT-qPCR Analysis

Total RNA was extracted using the method described above and then treated with RNase-free DNase I (Promega, Madison, WI, USA) to eliminate genomic DNA. According to the manufacturer’s instructions, total RNA (4 µg) was used for cDNA synthesis with oligo (dT) primer using M-MLV reverse transcriptase (Promega, Madison, WI, USA). RT-qPCR was carried out using SYBR Green Real-time PCR Master Mix (Toyobo, Osaka, Japan) with a Bio-Rad CFX96 Real-Time Detection System. For each gene detected by RT-qPCR, three biological replicates were analyzed. In each RT-qPCR run, 18S rRNA was used to normalize mRNA levels. Quantitative variations in different replicates were calculated using the delta-delta threshold cycle relative quantification method. The primers used for RT-qPCR were listed in Table S8.

### Aniline Blue Staining and Microscopy

After pollination, silk tissues were fixed in ethanol:chloroform:acetic acid = 6∶3:1 (v/v/v) overnight at 4°C. Then, they were washed twice in ddH_2_O and transferred into 8 M sodium hydroxide solutions for 1 to 2 h. Subsequently, the samples were re-rinsed twice in ddH_2_O and dried on filter paper before being stained with 1% aniline blue solution (Sigma, St. Louis, MO, USA) prepared with 0.1 M potassium phosphate buffer. The samples were stained for at least 12 h before photography. Both bright-field and epifluorescence imaging were performed on an Olympus BX51 microscope (Tokyo, Japan).

## Supporting Information

Table S1 **Distribution of reads sequenced from maize silk tissues before and after pollination in maize reference genome and reference gene database.** (A) Summary of reads mapped to reference genome. (B) Summary of reads mapped to reference gene database.
(DOC)

Table S2 **Genes expressed at four developmental stages of maize silk.**
(XLS)

Table S3 **Genes differentially expressed before and after pollination.**
(XLS)

Table S4 **Lists of differentially expressed genes with specific expression patterns.** (A) Genes expressed in IMS but absent from MS. (B) Genes expressed in MS but absent from IMS. (C) Genes which were induced by pollination.
(XLS)

Table S5 **Distribution of differentially expressed genes in 12 clusters.**
(XLS)

Table S6 **Conserved genes shared in both our SDEG dataset and those identified from previous microarray data.**
(XLS)

Table S7 **GO analysis of genes differentially expressed in both maize silk and** ***Arabidopsis*** **pistil before and after pollination.**
(DOC)

Table S8 **Primers used for RT-qPCR.**
(DOC)
