# Supplementary material for: Transcript Profile Analyses of Maize Silks Reveal Effective Activation of Genes Involved in Microtubule-Based Movement, Ubiquitin-Dependent Protein Degradation, and Transport in the Pollination Process
Source: PLoS One. 2013 Jan 3;8(1):e53545. doi: 10.1371/journal.pone.0053545 (PMC3536752; doi:10.1371/journal.pone.0053545)
Supplement: Table S1 — Distribution of reads sequenced from maize silk tissues before and after pollination in maize reference genome and reference gene database. (A) Summary of reads mapped to reference genome. (B) Summary of reads mapped to reference gene database. (DOC) [file pone.0053545.s001.doc]

**Table S1. Distribution of reads sequenced from maize silk tissues before and after pollination in maize reference genome and reference gene database.** (A) Summary of reads mapped to reference genome. (B) Summary of reads mapped to reference gene database.

(A)

|  | IMS | | MS | | 20MAP | | 3HAP | |
| --- | --- | --- | --- | --- | --- | --- | --- | --- |
|  | reads number | percentage | reads number | percentage | reads number | percentage | reads number | percentage |
| Total Reads | 7145682 | 100% | 6145170 | 100.00% | 7374812 | 100% | 7092051 | 100% |
| Total BasePairs | 350138418 | 100% | 301113330 | 100.00% | 361365788 | 100% | 347510499 | 100% |
| Total Mapped Reads | 5633214 | 78.83% | 4249353 | 69.15% | 5568786 | 75.51% | 4699095 | 66.26% |
| perfect match | 4114669 | 57.58% | 3090125 | 50.29% | 4074354 | 55.25% | 3441773 | 48.53% |
| <=3bp mismatch | 1518545 | 21.25% | 1159228 | 18.86% | 1494432 | 20.26% | 1257322 | 17.73% |
| unique match | 4780709 | 66.9% | 3666501 | 59.66% | 4713043 | 63.91% | 4008620 | 56.52% |
| multi-position match | 852505 | 11.93% | 582852 | 9.48% | 855743 | 11.6% | 690475 | 9.74% |
| Total Unmapped Reads | 1512468 | 21.17% | 1895817 | 30.85% | 1806026 | 24.49% | 2392956 | 33.74% |

(B)

|  | IMS | | MS | | 20MAP | | 3HAP | |
| --- | --- | --- | --- | --- | --- | --- | --- | --- |
|  | reads number | percentage | reads number | percentage | reads number | percentage | reads number | percentage |
| Total Reads | 7145682 | 100% | 6145170 | 100.00% | 7374812 | 100% | 7092051 | 100% |
| Total BasePairs | 350138418 | 100% | 301113330 | 100.00% | 361365788 | 100% | 347510499 | 100% |
| Total Mapped Reads | 5583189 | 78.13% | 4250779 | 69.17% | 5466562 | 74.12% | 4635615 | 65.36% |
| perfect match | 4251153 | 59.49% | 3214764 | 52.31% | 4149722 | 56.27% | 3515246 | 49.57% |
| <=2bp mismatch | 1332036 | 18.64% | 1036015 | 16.86% | 1316840 | 17.86% | 1120369 | 15.8% |
| unique match | 5062869 | 70.85% | 3868626 | 62.95% | 4922137 | 66.74% | 4190615 | 59.09% |
| multi-position match | 520320 | 7.28% | 382153 | 6.22% | 544425 | 7.38% | 445000 | 6.27% |
| Total Unmapped Reads | 1562493 | 21.87% | 1894391 | 30.83% | 1908250 | 25.88% | 2456436 | 34.64% |
