# Supplementary material for: Transcript Profile Analyses of Maize Silks Reveal Effective Activation of Genes Involved in Microtubule-Based Movement, Ubiquitin-Dependent Protein Degradation, and Transport in the Pollination Process
Source: PLoS One. 2013 Jan 3;8(1):e53545. doi: 10.1371/journal.pone.0053545 (PMC3536752; doi:10.1371/journal.pone.0053545)
Supplement: Table S7 — GO analysis of genes differentially expressed in both maize silk and Arabidopsis pistil before and after pollination. (DOC) [file pone.0053545.s007.doc]

**Table S7. GO analysis of genes differentially expressed in both maize silk and *Arabidopsis* pistil before and after pollination.**

| GO Term^a^ | Term type^a^ | Query item^b^ | Query total^c^ | Bg item^d^ | Bg total^e^ | P value^f^ | FDR^g^ |
| --- | --- | --- | --- | --- | --- | --- | --- |
| DNA replication initiation | P | 7 | 326 | 24 | 39203 | 9.10E-10 | 7.50E-07 |
| DNA-dependent DNA replication | P | 7 | 326 | 32 | 39203 | 8.40E-09 | 3.40E-06 |
| L-phenylalanine catabolic process | P | 5 | 326 | 19 | 39203 | 4.40E-07 | 9.00E-05 |
| lipid metabolic process | P | 27 | 326 | 1068 | 39203 | 1.10E-06 | 0.00016 |
| amine catabolic process | P | 6 | 326 | 51 | 39203 | 4.60E-06 | 0.00037 |
| carboxylic acid catabolic process | P | 6 | 326 | 55 | 39203 | 7.20E-06 | 0.00046 |
| L-phenylalanine metabolic process | P | 5 | 326 | 34 | 39203 | 9.40E-06 | 0.00055 |
| oxidation reduction | P | 47 | 326 | 2743 | 39203 | 1.30E-05 | 0.00069 |
| lipid biosynthetic process | P | 15 | 326 | 461 | 39203 | 1.30E-05 | 0.00069 |
| glutamine family amino acid biosynthetic process | P | 6 | 326 | 65 | 39203 | 1.90E-05 | 0.00086 |
| carbohydrate metabolic process | P | 31 | 326 | 1828 | 39203 | 0.00035 | 0.01 |
| two-component signal transduction system (phosphorelay) | P | 5 | 326 | 92 | 39203 | 0.0011 | 0.026 |
| intramolecular lyase activity | F | 7 | 326 | 22 | 39203 | 4.60E-10 | 2.20E-07 |
| ammonia-lyase activity | F | 5 | 326 | 24 | 39203 | 1.50E-06 | 0.0003 |
| cofactor binding | F | 26 | 326 | 1034 | 39203 | 1.90E-06 | 0.0003 |
| oxidoreductase activity | F | 55 | 326 | 3266 | 39203 | 5.00E-06 | 0.00061 |
| iron ion binding | F | 25 | 326 | 1187 | 39203 | 5.10E-05 | 0.0041 |
| transaminase activity | F | 5 | 326 | 57 | 39203 | 0.00012 | 0.0074 |
| carbon-nitrogen lyase activity | F | 5 | 326 | 57 | 39203 | 0.00012 | 0.0074 |
| coenzyme binding | F | 17 | 326 | 734 | 39203 | 0.00024 | 0.013 |
| two-component response regulator activity | F | 5 | 326 | 75 | 39203 | 0.00044 | 0.017 |

GO terms with P value<0.001 and FDR≤0.05 were regarded as overrepresented terms. ^a^GO term classifications: P, Biological Process; C, Cellular Component; F, Molecular Function. ^b^The distribution of query genes in each GO term. ^c^Total number of query genes with GO annotation in agriGO. ^d^The distribution of all the genes identified in maize genome version 5a in each GO term. ^e^Total number of genes with GO annotation in maize genome version 5a. ^f^Determined by Fisher exact test. ^g^Determined by Benjamini–Hochberg–Yekutieli procedure.
