# Supplementary material for: Transcript Profile Analyses of Maize Silks Reveal Effective Activation of Genes Involved in Microtubule-Based Movement, Ubiquitin-Dependent Protein Degradation, and Transport in the Pollination Process
Source: PLoS One. 2013 Jan 3;8(1):e53545. doi: 10.1371/journal.pone.0053545 (PMC3536752; doi:10.1371/journal.pone.0053545)
Supplement: Table S8 — Primers used for RT-qPCR. (DOC) [file pone.0053545.s008.doc]

**Table S8. Primers used for RT-qPCR.**

| Description | Name | Primer sequence |
| --- | --- | --- |
| Ras-related protein RIC2 | GRMZM2G127648-RL | GTATCACCACCACCAGAAC |
|  | GRMZM2G127648-RR | GAGGCGGCAATCAAGTAG |
| Histone H3 | GRMZM2G451254-RL | CCGATTAAGTCCTAGCCAATAG |
|  | GRMZM2G451254-RR | CGTGATGGGATGGGTATTTG |
| Beta-5-tubulin | GRMZM2G133802-RL | CTCAAGTTATGCGGTATGTAGTAG |
|  | GRMZM2G133802-RR | TGTCATAGGAAGGAACCATCAC |
| MFS18 protein | GRMZM2G105587-RL | CACACCACCGCTCCACAC |
|  | GRMZM2G105587-RR | ACATCGCCATCGCCATCG |
| Cyclin B2 | GRMZM2G138886-RL | CCAGTCCAGTCCTCCTCCTTC |
|  | GRMZM2G138886-RR | AATAACAAGCAGCAACGGTAACG |
| Ubiquitin-conjugating enzyme X | GRMZM2G002830-RL | ACTCGCCGCTCAACACTC |
|  | GRMZM2G002830-RR | CGCTGTCATCCTCCTCTACG |
| Brassinosteroid insensitive 1-associated receptor kinase 1 | GRMZM2G015933-RL | CACTGATGGCGACATACTG |
|  | GRMZM2G015933-RR | CGGATGACGGAGTTGTTC |
| Endo-1,4-beta-xylanase | GRMZM2G031004-RL | CTAAGCCAGCAGCCACAG |
|  | GRMZM2G031004-RR | TCTCCTCCTCAGCGAACG |
| Xylanase inhibitor protein 1 | GRMZM2G447795-RL | CAAGGAGGAGGGCACACTG |
|  | GRMZM2G447795-RR | CACGCTGTAGAAGGAGATGATG |
| Oligopeptide transporter 4 | GRMZM2G112456-RL | CATTGCCGCCACCATCAC |
|  | GRMZM2G112456-RR | CGCCATCAACGAGTAGAACC |
| AGAMOUS-like protein | GRMZM2G359952-RL | CCACCACCACCACCACAC |
|  | GRMZM2G359952-RR | GCCACCTGCTCCTTGACC |
| MADS box protein | GRMZM2G003514-RL | CACTCAGTTGCTATGGAC |
|  | GRMZM2G003514-RR | GATGGATGGATTAACAGAAG |
| Ubiquitin-conjugating enzyme protein E2 | GRMZM2G012052-RL | CCAAAACCCTAGCCCTGCTT |
|  | GRMZM2G012052-RR | TATAGGGTGTCAGGCGGTCC |
| EREBP-4 like protein | GRMZM2G071844-RL | GCGAACGATTACCTCTACTG |
|  | GRMZM2G071844-RR | GGCTCCAAACCCATATTCC |
| S-domain class receptor-like kinase 3 | GRMZM2G309025-RL | TCTATTGTGGCATACTTCC |
|  | GRMZM2G309025-RR | AATGGCTGTTGAATCTGG |
| Ubiquitin-conjugating enzyme E2 | GRMZM2G007381-RL | GCTTACCTTGCTGTGGTGGT |
|  | GRMZM2G007381-RR | GCAGACTGATACGAAGGCACA |
| 17.4 kDa class I heat shock protein 3 | GRMZM2G335242-RL | TCGTTGTGCCCAAGGAAG |
|  | GRMZM2G335242-RR | TACACTCTGCCACTCATCG |
| Fructose-2, 6-bisphosphatase | GRMZM2G127598-RL | CCACCGCTGCTCTCGTCTG |
|  | GRMZM2G127598-RR | GCCCTCCCACTGACCTTGAC |
| Histone H4 | GRMZM2G479684-RL | GCGGTCGTGTTGTGTTCTTG |
|  | GRMZM2G479684-RR | ATTACAGCAGCGAGATATTACAGC |
| Embryogenesis-associated protein EMB8 | GRMZM2G005444-RL | CTTCATTCACATACAAGAGACAG |
|  | GRMZM2G005444-RR | CAGGAACCACAACTACAAGG |
| Hexokinase | GRMZM2G432801-RL | GAGCATTACAAGAAGTTCAG |
|  | GRMZM2G432801-RR | TAGTCACTCTCGCCATAC |
| Pollen signalling protein with adenylyl cyclase activity | GRMZM2G060583-RL | CTCATCCTCCTCGCTTCTG |
|  | GRMZM2G060583-RR | TTGCCACTTCCTCGTTCC |
| Bowman-Birk serine protease inhibitor | GRMZM2G075315-RL | TTGGTGGTTCGGTGGCTAATC |
|  | GRMZM2G075315-RR | CTTGCTTGTGGTAGGTTCTAATGG |
| Gibberellin 20 oxidase 2 | GRMZM2G099467-RL | TCGGATACGGATGGATGGATAC |
|  | GRMZM2G099467-RR | GACCACAGCCTTGATTGATACC |
| Potasium ion uptake permease 1 | GRMZM2G084779-RL | TAGCATTCCAGAGCATAGG |
|  | GRMZM2G084779-RR | CCATCACCGTTATCATTAGC |
| bZIP transcription factor 1 | GRMZM2G117851-RL | CATCAGACCAAGCACAAG |
|  | GRMZM2G117851-RR | CCATCCATCATCCAGAGG |
